# A Highly Selective Sensor for Cyanide in Organic Media and on Solid Surfaces

**DOI:** 10.3390/s16030271

**Published:** 2016-02-24

**Authors:** Belygona Barare, Ilknur Babahan, Yousef M. Hijji, Enock Bonyi, Solomon Tadesse, Kadir Aslan

**Affiliations:** 1 Department of Chemistry, Morgan State University, Baltimore, MD 21251, USA; bebar1@morgan.edu (B.B.); ilknurbabahan@yahoo.com (I.B.); enbon1@morgan.edu (E.B.); solomon.tadesse@morgan.edu (S.T.); 2 Department of Chemistry, Adnan Menderes University, Aydin 09010, Turkey; 3 Department of Chemistry and Earth Sciences, Qatar University, P.O. Box 2713, Doha, Qatar; yousef.hijji@qu.edu.qa

**Keywords:** IR-786, anion sensing, cyanide sensing, cyanine dyes, near-infrared fluorophores, optical sensors, chemical sensors

## Abstract

The application of IR 786 perchlorate (IR-786) as a selective optical sensor for cyanide anion in both organic solution (acetonitrile (MeCN), 100%) and solvent-free solid surfaces was demonstrated. In MeCN, IR-786 was selective to two anions in the following order: CN^−^ > OH^−^. A significant change in the characteristic dark green color of IR-786 in MeCN to yellow was observed as a result of nucleophilic addition of CN^−^ to the fluorophore, *i.e.*, formation of IR 786-(CN), which was also verified by a blue shift in the 775 nm absorbance peak to 430 nm. A distinct green fluorescence emission from the IR-786-(CN) in MeCN was also observed, which demonstrated the selectivity of IR-786 towards CN^−^ in MeCN. Fluorescence emission studies of IR-786 showed that the lower detection limit and the sensitivity of IR-786 for CN^−^ in MeCN was 0.5 μM and 0.5 to 8 μM, respectively. The potential use of IR-786 as a solvent-free solid state sensor for the selective sensing and monitoring of CN^−^ in the environment was also demonstrated. On solvent-free solid state surfaces, the sensitivity of the IR-786 to CN^−^ in water samples was in the range of 50–300 μM with minimal interference by OH^−^.

## 1. Introduction

Detection of anions in biological systems and the environment within the desired concentration range in a timely manner is central to federal and state regulations imposed on commercial chemical and biological processes [1,2,3,4,5]. Among the common anions are cyanide, hydroxide, phosphate and fluoride; while cyanide is an important industrial chemical, it is known to be extremely toxic to human and animals even when present in insignificant concentrations between 0.5 and 3.5 mg per kilogram of body weight [3]. Cyanide is more lethal in two forms: free cyanide and hydrogen cyanide (HCN) [6,7]. Cyanide anion (CN^−^) has the detrimental impact of forming complexes in the cell mitochondrion, interfering with the capacity of a cell to produce energy, consequently leading to accumulation of chemicals in the bloodstream [6,7]. Consequently, the U.S. Environmental Protection Agency (EPA) has set a CN^−^ limit in drinking water of 2 ppm (76 μM) [7].

Conventional methods of testing for cyanide such as titration, distillation, chromatography or potentiometry are expensive, laborious and time consuming [5]. Therefore, there is still a need to develop more robust, inexpensive and less laborious testing methodologies for cyanide [5]. In an effort to make anion quantitative detection and monitoring inexpensive, easy and straight-forward, a number of colorimetric detection methods were developed [7,8,9]. For cyanide detection, these sensors rely on either displacement or a chemodosimeter principle [7]. Some of these sensors are synthesized in several steps, which could result in the escalation of the overall costs as well as environmental pollution [10]. In addition, several of the available cyanide sensors are limited as they work best in organic solvents, suffer interference by other anions (OH^−^, F^−^ or AcO^−^), and/or have low sensitivity due to their short absorption and emission wavelength, which interferes with other natural processes [9,11]. For example, Isaad *et al.* have developed a new chemodosimeter-sensitized starch film [12], cellulose [13] and water soluble and fluorescent copolymers [14]. Immersion of these functionalized films in an aqueous solution of cyanide induced a color change that can be used for the detection of cyanide. These easy-to-use materials also showed also a high degree of cyanide selectivity in aqueous media [12,13,14].

Near-infra red (NIR) chemical sensors in the form of cyanine dyes, independent of their operational principle, have the potential to overcome these difficulties by employing long-wavelength absorbance, large extinction coefficients and emission spectra in the visible to near infrared region [9,11]. These dyes have found use as additives to photographic emulsions, laser dyes, optical recording media, solar cells and biological applications such as non-invasive alternatives to radionuclides [11,15,16,17]. In addition, these dyes can be synthesized and purified by simple methods that do not rely on the use of excessive solvents, such as microwave and solid phase protocols [15,18,19,20]. These developments have fueled the development of new NIR dyes with desirable photophysical properties as well as synthesized on a large scale [15,17]. NIR chemical sensors such as IR-786 operate in a longer wavelength range (absorption peak between 700 and 900 nm) [21], and exhibit minimal background interferences affording for high sensitivity for anions compared to the short wavelength organic sensors [17,20,21,22]. Moreover, these dyes are relatively inexpensive and can operate in both aqueous and organic media [9,18,19]. For example, a naturally occurring dye (*i.e.*, 2-hydroxy-1,4-naphthoquinone or lawsone) was used to detect acetate and cyanide in aqueous media [23]. However, lawsone operates in the short-wavelength (visible) region, is prone to background interferences and scattering [9] and therefore is not best suited for biological applications [22,24].

In this work, we demonstrate the use of a cyanine dye, IR-786, which operates in the NIR region, as an anion sensor for the qualitative and quantitative sensing of cyanide in water. IR-786 has a heptamethine bridge with a peak absorption and emission in the range of 760–780 nm and 795–815 nm, respectively [21]. We demonstrated that IR-786 can detect CN^−^ in 100% MeCN with interference from OH^−^ and CN^−^ on solvent-free solid surfaces in a selective manner using absorbance and fluorescence spectroscopy. The proposed chemical sensor, IR-786, is capable of exhibiting NIR absorptions as well emission in both organic and aqueous media, properties not common to other anion probes.

## 2. Materials and Methods

### 2.1. Materials and Instrumentation

All anions, in the form of sodium or tetrabutylammonium salts, all solvents and IR 786 dye, 98% (Catalog No: 102185035) were purchased from Sigma-Aldrich Chemical Company (St. Louis, MO, USA) and used without further purification. Solid surfaces (chromatography paper, 3 mm, Catalog Number: 3030–6187) were purchased from VWR International Ltd. (Bridgeport, NJ, USA).

All UV/vis spectroscopy experiments were carried out in solution phase recorded using a Cary UV/vis-NIR spectrophotometer 5000 (Varian, Walnut Creek, CA, USA) equipped with a quartz cuvette (path length = 1 cm). Fluorescence emission spectra experiments were measured using a Cary 60 series spectrometer (Agilent, Walnut Creek, CA, USA). Spectrophotometric titrations were performed at various concentrations of the sensors in MeCN and water: MeCN mixtures. The ^1^H- and ^13^C-NMR spectra were recorded using an Avance™ 400 MHz spectrometer (Bruker, Billerica, MA, USA). FT-IR spectra were obtained from an Agilent Cary 630 FTIR spectrometer and reported in cm^−1^ units.

### 2.2. Preparation of IR-786 Stock Solutions

Stock solutions of IR-786 (50 μM) (100:0, 75:25, 50:50, 25:75 = H_2_O/MeCN) and organic (100% MeCN) media were prepared at room temperature. Aliquots of fresh sodium and tetrabutylammonium salt standard solutions of the anions were added to the IR-786 stock solutions, mixed and the UV/vis and fluorescence spectra of the mixture were recorded as described in the following sections.

### 2.3. Qualitative Response of IR-786 to Anions in Solution Phase and on Solid Surfaces

Solution phase experiments were carried out by addition of 1.0 mL of anions from tetrabutylammonium salts (up to 50 μM in MeCN) to 1.0 mL of IR-786 (50 μM in MeCN). Solid surfaces were cut into 6 mm pieces and incubated in IR-786 in MeCN until the surfaces were completely dry at room temperature. A drop (20 μL) of anion solutions from tetrabutylammonium salts (CN^−^ and OH^−^ up to 1000 μM in deionized water) was incubated on the dry solid surfaces with dried IR-786 for 2 min at room temperature. Color changes in solution phase and on solid surfaces were observed visually under normal light and under a hand held UV lamp (λ = 365 nm) upon addition of various anions at room temperature.

### 2.4. Quantitative Response of IR-786 to Anions in Solution Phase and on Solid Surfaces

Standard solutions of IR-786 (5.0 μM) and sodium/tetrabutylammonium salt solutions (1.0 × 10^−2^ M) of the anions (F^−^, AcO^−^, H_2_PO_4_^−^, Br^−^, Cl^−^, ClO_4_^−^ and HSO_4_^−^) were prepared in MeCN and in aqueous media. We note that the concentration of IR-786 was reduced to 5.0 μM to obtain a maximum absorbance value less than 1.0 and fluorescence spectroscopy studies were carried out using the identical concentrations for the sake of consistency. The UV-visible titrations were carried out by adding up to 8.0 or 10 μM of selective anions to 2.0 mL solution of IR-786 solution (Note: the total volume of anions added was less than 3.0 μL). After mixing for a few seconds, UV-vis, fluorescence spectra of the samples were recorded. Modifications of UV-vis or fluorescence spectra data were processed through a plot of inverse changes in absorbance/emission band against the inverse of anion concentration. Binding constants were determined by use of Benesi-Hildebrand plots [25] and reported as an average of at least three trials. In this regard, IR-786 (5.0 μM) and each selective anions (5.0 μM) were mixed in the ratio of 1:9, 2:8, 3:7, 4:6, 5:5, 6:4, 7:3, 8:2, 9:1 in MeCN. After mixing for a few seconds, UV-vis spectra of the samples were recorded at room temperature.

### 2.5. Characterization of IR-786-(CN) Complex Using ^1^H-NMR

A stock solution of IR-786 (5.0 μM) and 1.0 mL of tetrabutylammonium cyanide (5.0 μM) was mixed and the color change from green to yellow was observed visually under normal light and under a hand held UV lamp (λ = 365 nm). The mixture was characterized using ^1^H-NMR and the summary of peaks are given below: ^1^H-NMR (400 MHz, CD_3_CN): δ = 8.40 (d; 1H), 7.53 (d; 1H), 7.46 (t; 1H), 7.40 (t; 1H), 7.11 (t; 1H), 7.07 (d; 1H), 6.85 (t; 1H), 6.74 (t; 1H), 6.52 (d; 1H), 6.21 (d; 1H), 5.90 (d; 1H), 5.49 (d; 1H), 3.17 (s; 3H), 3.11 (s; 3H), 2.68 (s; 3H), 2.22 (s; 3H), 1.72 (s; 3H), 1.27 (s; 3H), 1.62 (quin; 2H), 1.36 (sext; 2H), 1.06 (d; 2H), and 0.99 (t; 3H).

### 2.6. Characterization of IR-786-(OH) Complex Using ^1^H-NMR

A stock solution of IR-786 (5.0 μM) and 1.0 mL of tetrabutylammonium hydroxide (5.0 μM) was mixed and the color change from green to yellow was observed visually under normal light and under a hand held UV lamp (λ = 365 nm). The mixture was characterized using ^1^H-NMR and the summary of peaks are given below: ^1^H-NMR (400 MHz, CD_3_CN): δ = 8.52 (s; 1H), 7.55 (d; 1H), 7.46 (d; 1H), 7.22 (t; 1H), 7.17 (t; 1H), 6.89 (t; 1H), 6.86 (t; 1H), 6.76 (d; 1H), 6.70 (d; 1H), 5.86 (d; 1H), 5.84 (d; 1H), 5.50 (d; 1H), 5.36 (d; 1H), 3.13 (s; 3H), 3.11 (s; 3H), 3.08 (s; 3H), 2.40 (s; 6H), 1.12 (s; 3H), 1.62 (quin; 2H), 1.36 (sext; 2H), 1.11 (d; 2H), and 0.99 (t; 3H).

### 2.7. Characterization of IR-786 in the Presence of Cyanide and Hydroxyl Anions IR-786-(CN)-(OH) Using ^1^H-NMR

A stock solution of IR-786 (5.0 μM), 1.0 mL of tetrabutylammonium cyanide and 1.0 mL of tetrabutylammonium hydroxide (5.0 μM) was mixed and the color change from green to yellow was observed visually under normal light and under a hand held UV lamp (λ = 365 nm). The mixture was characterized using ^1^H-NMR and the summary of peaks are given below: ^1^H-NMR (400 MHz, CD_3_CN): δ = 8.54 (d; 1H), 7.53 (d; 1H), 7.46 (t; 1H), 7.40 (t; 1H), 7.11 (t; 1H), 7.07 (d; 1H), 6.85 (t; 1H), 6.74 (t; 1H), 6.52 (d; 1H), 6.21 (d; 1H), 5.90 (d; 1H), 5.49 (d; 1H), 3.17 (s; 3H), 3.11 (s; 3H), 2.68 (s; 3H), 2.22 (s; 3H), 1.72 (s; 3H), 1.27 (s; 3H), 1.62 (h; 2H), 1.36 (quin; 2H), 1.06 (sext; 2H), and 0.99 (t; 3H).

## 3. Results

**Scheme 1:**
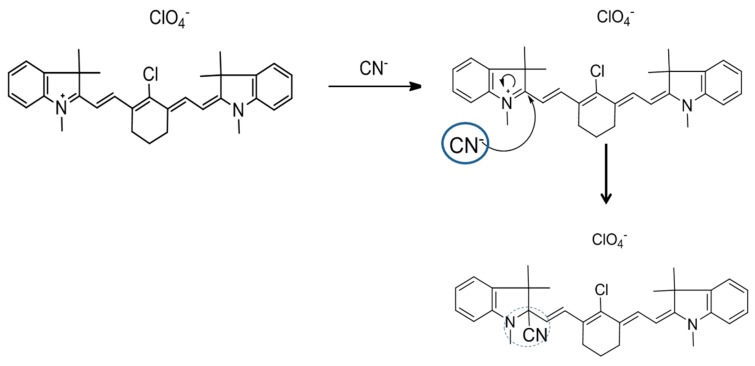
Schematic depiction of the nucleophilic addition of CN^−^ to IR-786.

IR-786 is a commercially available cyanine dye, traded as IR 786 perchlorate (2-(2-[2-chloro-3-([1,3-dihydro-1,3,3-trimethyl-2*H*-indol-2-ylidene]ethylidene)-1-cyclohexen-1-yl]ethenyl)-1,3,3-tri-methylindolium perchlorate) with a symmetrical heptamethine bridge (Scheme 1) [21]. IR-786 possesses useful characteristics to be considered as an anion sensor: absorption spectral near-infra red region, Stokes’ shift (25 nm), large extinction coefficient, high quantum yields [16] and ability to undergo conjugation with selective anions [9,22]. Therefore, we investigated whether IR-786 can be used as an anion sensor by observing the changes in its color, absorption and emission spectrum before and after the addition of anions (F^−^, AcO^−^, CN^−^, OH^−^, H_2_PO_4_^−^, Br^−^, Cl^−^, N_3_^−^, ClO_4_^−^ and HSO_4_^−^) in organic media and solvent-free solid surfaces.

### 3.1. Qualitative Assessment of Selectivity of IR-786 for Anions in Organic (MeCN) Media

**Figure 1:**
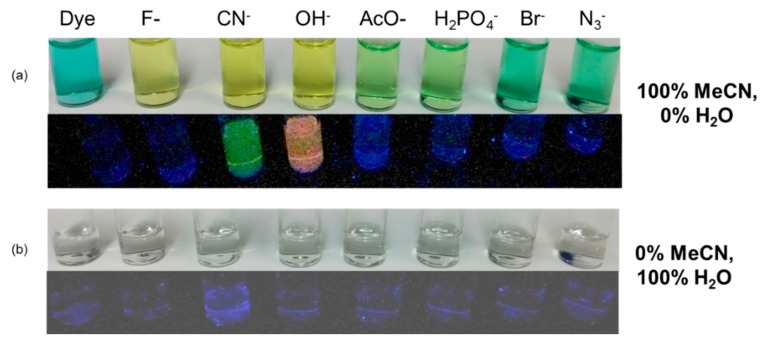
(**a**) Color changes of IR-786 (5 μM) under normal light (top) and under hand held UV lamp (λ = 365 nm) in (**a**) 100% MeCN and (**b**) aqueous solution (100% H_2_O) upon the addition of various anions.

**Figure 2:**
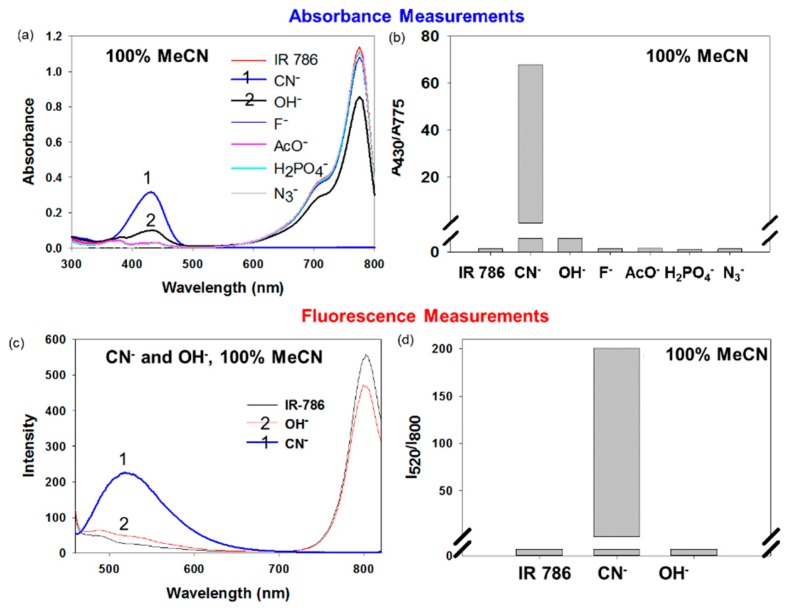
(**a**) UV-vis absorption spectra and (**b**) absorbance ratiometric values (absorbance value at 430 divided by absorbance value at 775 nm) before and after the addition of 1.0 equivalence of various anions to IR-786 (5 μM) in 100% MeCN and (**c**) emission spectra and (**d**) emission ratiometric values (intensity value at 520 nm divided by intensity value at 800 nm) obtained from (**a**).

Upon addition of 1.0 equivalents of all the studied anions to IR-786, only three anions produced a discernable color change from green to yellow as observed by the naked eye: in descending order of color changes intensity, these anions were CN^−^, OH^−^ and F^−^ (Figure 1 and Figure S1). The addition of AcO^−^ and H_2_PO_4_^−^ resulted in slight discoloration in the IR-786 solution in MeCN and there were no noticeable color changes in the other anions tested (Br^−^, Cl^−^, ClO_4_^−^, HSO_4_^−^). Upon exposure of IR-786 solution after the addition of the anions to a UV light at 365 nm, CN^−^ and OH^−^ resulted in a green and orange emission, respectively. No color change were observable for all other anions (Figure 1 and Figure S1). The observed color changes (in Figure 1 and Figure S1) were supported by the absorption spectra as shown in Figure 2a, where IR-786 showed selectivity towards CN^−^ and OH^−^ in terms of appearance of an additional absorbance peak at 430 nm (yellow color). The selectivity of IR-786 towards CN^−^ was further confirmed by ratiometric plot of the absorbance values of the two prominent peaks at 430 nm and 775 nm (Figure 2a), where the ratiometric value for CN^−^ is ~10 fold larger than the value for OH^−^. There were no significant spectral changes for IR-786 upon the addition of Br^−^, Cl^−^, N_3_^−^, ClO_4_^−^ and HSO_4_^−^.

In order to investigate whether the presence of water in the solution can affect the sensitivity and selectivity of the IR-786 towards anions, several water– MeCN mixtures (100:0, 75:25, 50:50, 25:75 water/MeCN) was used as the sensor medium. The most noticeable visual change in the IR-786 was observed with the 25:75 water/MeCN mixture (Figures S1 and S2). The increase in the water content of the IR-786 solution result in no color change, which can be partly attributed to the decrease in the solubility of IR-786 in the mixture due to the increased water content (IR-786 is not soluble in water or mixture less than 75%:25% MeCN:water).

In addition to visual observations of the color change in the IR-786 solution with the addition of anions, qualitative assessment of anion response of IR-786 to anions was evaluated by fluorescence emission spectroscopy. When illuminated with a hand held UV lamp at 365 nm, the addition of CN^−^ and OH^−^ to IR-786 solutions resulted in green and orange fluorescence emission from IR-786 solution in 100% MeCN and 25:75 water–MeCN, respectively (Figure 1 and Figure S1). The addition of other anions (F^−^, AcO^−^, H_2_PO_4_^−^, Br^−^, Cl^−^, OH^−^, N_3_^−^) did not result in fluorescence emission.

To further investigate the fluorescence-based response of IR-786 solution to anions, fluorescence spectra of the mixtures were measured between 460 nm to 820 nm (Figure 2b). IR-786 in MeCN solutions show a single peak 800 nm, which was completely quenched after the addition of CN^−^ and slightly decreased by the addition of OH^−^. On the other hand, a new emission peak at 520 nm was observed after the addition of CN^−^ in conjunction with the disappearance of the peak at 800 nm. The ratiometric plot of the fluorescence emission values of the two prominent peaks at 520 nm and 800 nm (Figure 2b), where the ratiometric values for CN^−^ is ~40 fold larger than the value for OH^−^. These qualitative observations imply that IR-786 shows selectivity to both anions in organic media, however, it is more selective and sensitive to CN^−^ than OH^−^. Subsequently, only CN^−^ and OH^−^ were used in the remainder of the study presented here. It is important to note that OH^−^ is regarded as an interference for the detection of CN^−^.

### 3.2. Quantitative Assessment of Selectivity of IR-786 for Anions in MeCN

**Figure 3:**
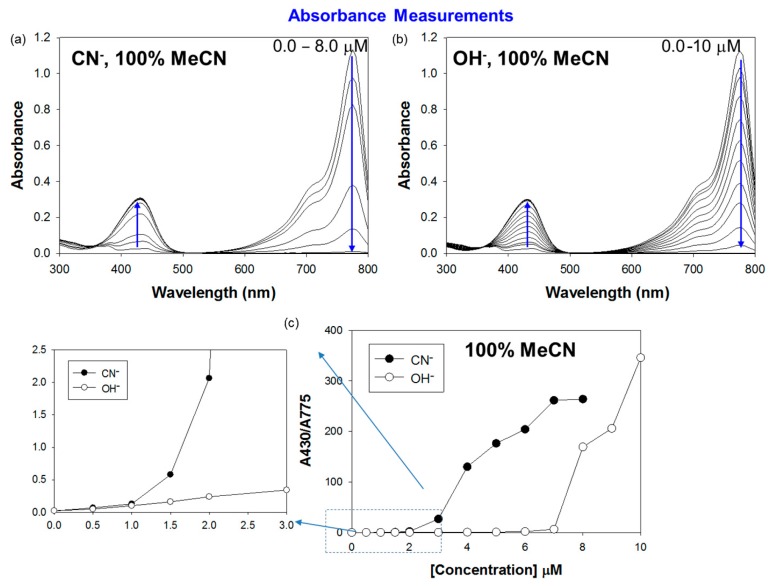
UV-vis absorption spectra of IR-786 in 100% MeCN (5 μM) before and after the addition of (**a**) CN^−^ (up to 8 μM) and (**b**) OH^−^ (up to 10 μM) and (**c**) absorbance ratiometric values (absorbance value at 430 divided by absorbance value at 775 nm) obtained from (**a**). Arrows show the direction of increased amount of anions.

**Figure 4:**
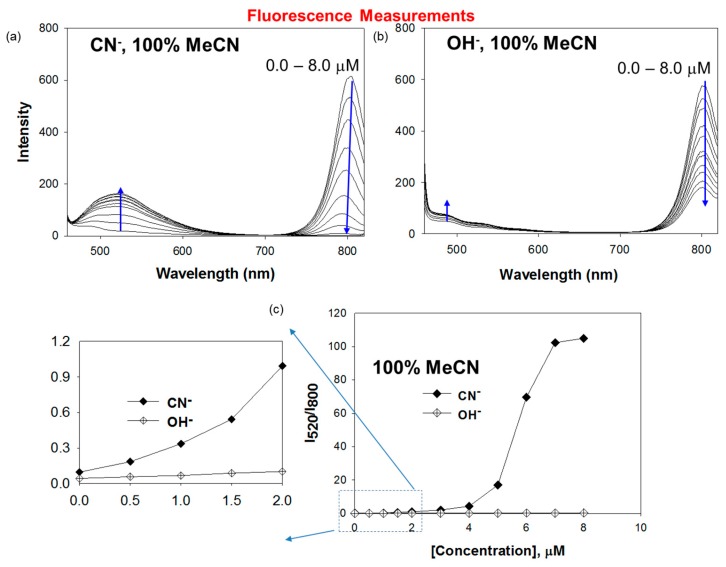
Fluorescence emission spectra (λex = 430 nm, excitation slit = 20 nm, emission slit = 20 nm), for IR-786 (50 μM) in 100% MeCN (**a**) CN^−^ (up to 8 μM) and (**b**) OH^−^ (up to 8 μM) and (**c**) fluorescence emission ratiometric values (intensity value at 520 nm divided by intensity value at 800 nm) obtained from (**a**). Arrows show the direction of increased amount of anions.

The selectivity of IR-786 to CN^−^ and OH^−^ in 100% MeCN and 25:75 water–MeCN mixture was further assessed in a quantitative manner using absorbance (Figure 3 and Figure S3a) and fluorescence measurements (Figure 4 and Figure S3b). Upon incremental addition of CN^−^ (0.0–8.0 μM) and OH^−^ (0.0–10 μM) (separate experiments), the absorbance band at 775 nm gradually decreased accompanied by a gradual increase in the new peak at 430 nm (Figure 3a). An isosbestic point at 550 nm indicated the formation of a new complex between IR-786 and the anions, *i.e.*, IR-786-(CN) or IR-786-(OH) (Figure 3 and Figure S3). Job’s plots displayed a 1:1 stoichiometric complex between IR-786 and CN^−^ (Figure S4), and IR-786 and OH^−^ (Figure S5). In addition, the UV-vis absorption spectra measurements were used to calculate the binding constants of CN^−^ and OH^−^ from the variation of absorbance at λ_max_ = 430 nm (Figure S6). These constants were found to be 2.7 × 10^5^ M^−1^ and 2.5 × 10^5^ M^−1^, for CN^−^ and OH^−^, respectively. We note the pH of IR-786 solution did not change the titration of stock solution of CN^−^ and OH^−^ in buffer (Figure S7).

In order to determine the detectable concentration range for CN^−^ and OH^−^ in solution, the ratiometric absorbance and fluorescence plots as a function of concentration was plotted (Figure 3b and Figure 4b) using the spectral data presented in Figure 3a and Figure 4a. The ratiometric absorbance plot (Figure 3b) shows that: (i) the addition of CN^−^ as low as 1.5 μM (lower detection limit) result in a detectable colorimetric response in MeCN, where the same level of ratiometric response is measured for OH^−^ as low as 4.0 μM and (ii) the detectable concentrations range for CN^−^ and OH^−^ was 1.5–6.0 μM and 7.0–10 μM, respectively. These observations imply that the colorimetric detection of CN^−^ in MeCN can be carried out for a narrow concentration range with strong interference from OH^−^, *i.e.*, similar ratiometric values are observed both anions. On the other hand, the ratiometric fluorescence-based detection of CN^−^ in MeCN by IR-786 is more promising due to the large ratiometric response for CN^−^ (up to 120) in the range of 0.5–7.0 μM and minimal interference by OH^−^ (the ratiometric response for OH^−^ was 3- to 500-fold less than that for CN^−^ at 5.0–45 μM). Similar results were obtained for IR-786 in 25:75 water–MeCN (Figure S3), where the interference by OH^−^ was markedly less in the ratiometric absorbance and fluorescence plots as compared to those observed in 100% MeCN. This observation can be attributed to the presence of OH^−^ in the water–MeCN mixture. Consequently, IR-786 can be used as an anion sensor in 25:75 water–MeCN.

### 3.3. Quantitative Response of IR-786 to Anions on Solid Surfaces

**Figure 5:**
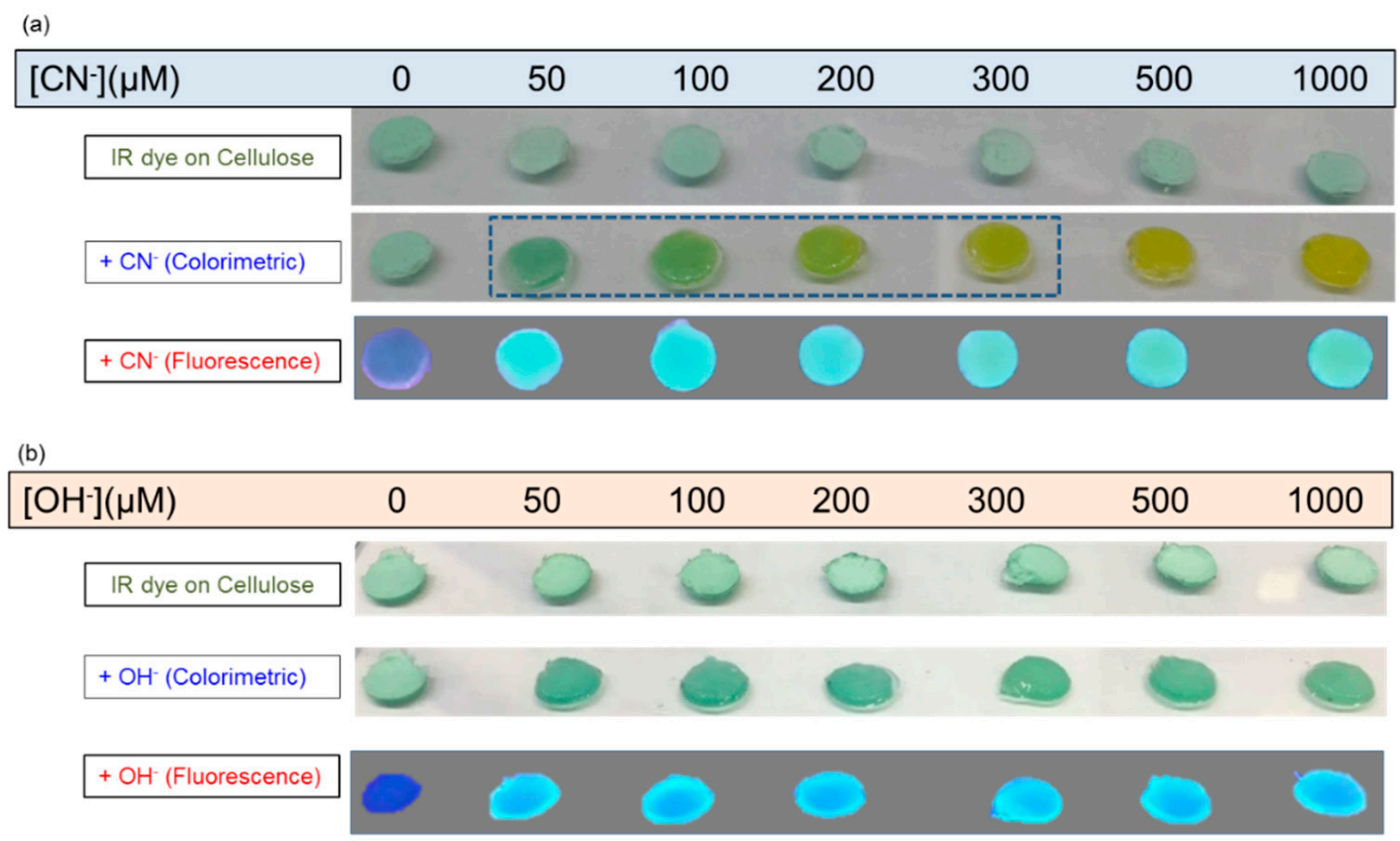
Real-color photographs of colorimetric and fluorescence emission from IR-786 dried on to solvent-free solid surfaces before and after the addition of (**a**) CN^−^ and (**b**) OH^−^.

The use of IR-786 as a solid-state sensor was also demonstrated. In this regard, the detectable concentration range for CN^−^ and OH^−^ on a solid surface with dried IR-786 was determined by colorimetric and fluorescence measurements (Figure 5). Figure 5a shows that the detectable range for CN^−^ in water sample by IR-786 solid state sensor is 50–300 μM: an increase in the concentration of CN^−^ resulted in a change in color of the sensor from the original green color (no CN^−^) to yellow-green (50.0–300 μM) and yellow (>500 μM^−^). Similar changes in the fluorescence emission was observed for CN^−^ in water sample: blue emission (no CN^−^), blue-green (50.0–300 μM) and green (>500 μM^−^). In addition, the potential interference by OH^−^ was also investigated. Figure 5b shows that the addition of OH^−^ (up to 1000 μM) on to the solid surface with dried IR-786 did not result in significant changes in color and fluorescence emission. These results demonstrate the utility of IR-786 dye as a selective CN^−^ sensor with minimal interference from OH^−^. We note that the sensitivity of IR-786 towards CN^−^ is lower than those novel chemical sensors reported in the literature [12,13,14]. However, our results show that IR-786 is effective in the detection of CN^−^ within the current limits set by EPA.

### 3.4. Elucidation of Binding Mechanism for Anions in MeCN

**Figure 6:**
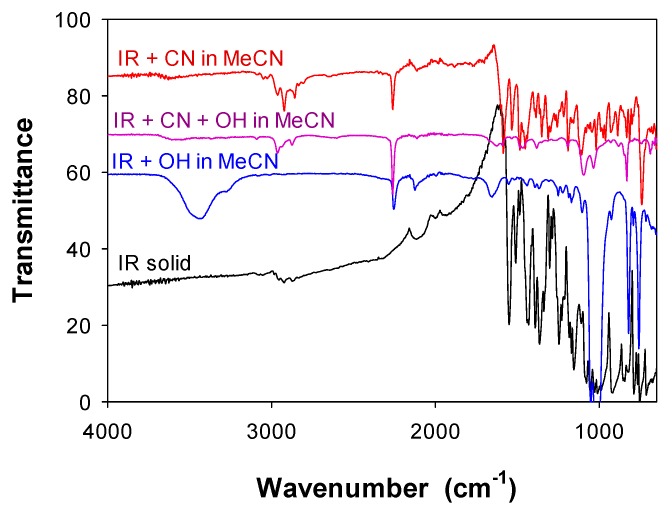
FT-IR spectra of IR-786 dye in different forms: solid as purchased, in MeCN and with CN^−^ alone, in MeCN and with OH^−^ alone and in MeCN and with OH^−^ and CN^−^.

It is important to understand the binding mechanisms between IR-786 and the anions to gain further insights to anion sensing applications of IR-786. In this regard, the characterization of IR-786 in the absence and presence of individual anions and both anions were carried out by FT-IR (Figure 6) and NMR spectroscopy (data presented in the Materials and Methods section). The FT-IR spectrum of solid IR-786 has characteristic peaks for aromatic groups between 2800 and 2980 cm^−1^ and lacks two important bands (–CN: 2200 cm^−1^ and OH: 3224 cm^−1^) due to the absence of these groups in the molecule [26,27]. A strong peak for –CN at 2200 cm^−1^ appeared when IR-786 is dissolved in MeCN and CN^−^ is added and the aromatic peaks at 2800–2990 cm^−1^ became stronger due to the conversion of aromatic rings to aliphatic groups. A broad peak for –OH at 3224 cm^−1^ was observed when only OH^−^ is added to IR-786, where –CN peak at 2200 cm^−1^ still exists (due to MeCN). The presence of both CN^−^ and OH^−^ in the same solution of IR-786 resulted in observation of all three characteristic peaks: strong –CN peak 2200 cm^−1^, less pronounced –OH peak 3224 cm^−1^ and less pronounced aromatic peaks at 2800–2990 cm^−1^. There are two possible explanations for these observations: (i) CN^−^ and OH^−^ binds to the different parts of IR-786 and/or (ii) a mixture of IR-786-(OH) and IR-786-(CN) can exist when both anions are present in the same IR-786 solution. In addition, the pH of the IR-786 solution did not change significantly, which implies the effect of pH on observed fluorescence emission was negligible. In order to further understand the binding mechanism for CN^−^ and OH^−^, ^1^H-NMR spectroscopy was employed.

The ^1^H-NMR spectrum of IR-786-(CN) indicates the presence of eight aromatic protons (δ_H_: 8.39, 7.53, 7.46, 7.40, 7.11, 7.07, 6.85 and 6.74), four CH protons (δ_H_: 6.52, 6.21, 5.90 and 5.49 as doublet), six –CH_3_ protons (δ_H_: 3.17, 3.11, 2.68, 2.22, 1.72 and 1.27) and four peaks for tetrabutylammonium cyanide (δ_H_: 1.62, 1.36, 1.06 and 0.99). The addition of CN^−^ to IR-786 resulted in disappearance of the polarized C=N bond of indolium group and the formation of a new C-N bond. As illustrated in Scheme 1, the reaction mechanism can be explained by the nucleophilic addition reaction of cyanide anion with the polarized C=N bond of the indolium group. These observations clearly indicated that the cyanide anion was added to the C=N group of IR-786 and suggested 1:1 reaction between IR-786 and CN^−^.

**Scheme 2:**
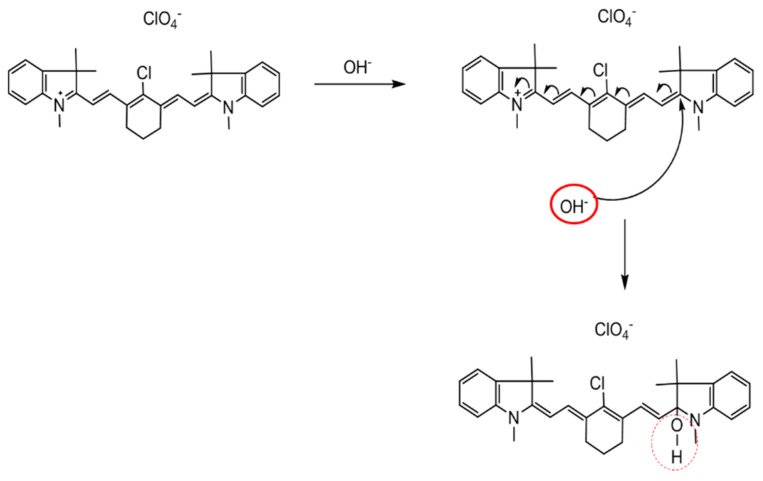
Schematic depiction of nucleophilic addition of OH^−^ to IR-786.

The spectrum of [IR-786-(OH)] shows eight aromatic protons (δ_H_: 7.53, 7.46, 7.22, 7.17, 6.89, 6.86, 6.76 and 6.70), four CH protons (δ_H_: 5.86, 5.84, 5.50 and 5.36 as doublet), five –CH_3_ protons (δ_H_ 3.13, 3.11, 3.08, 2.40 and 1.12) and four peaks for tetrabutylammonium hydroxide (δ_H_: 1.62, 1.36, 1.11 and 0.99). The signals for–OH bonds of IR-786-(OH) and tetrabutylammonium hydroxide are observed at 8.52 ppm and 7.21 ppm as a singlet, respectively. As illustrated in Scheme 2, OH^−^ anion was added to the indole group and formed a new C-OH bond. An intramolecular charge transfer from the indole group to indolium group of [IR-786-(OH)] occurs as a result of π-delocalization from the indole group.

The main difference in the ^1^H-NMR spectrum of IR-786-(OH) with that of IR-786-(CN) is the presence of the hydroxyl proton signal at δ_H_ 8.32 (1H, s) for IR-786-(OH) and a shift in the signals of aromatic protons to up field. The chemical shifts for aromatic protons show that CN^−^ an OH^−^ ions are bound to different carbon atoms. Subsequently, IR-786-(OH) compound displays an intramolecular charge transfer from the indole group to indolium group, which is supported by the change in color of the solution from green to yellow as observed by colorimetric measurements. In addition, the ^1^H-NMR spectra of IR-786-(CN)-(OH) and IR-786-(CN) are very similar: the peaks of aromatic protons of IR-786-(CN)-(OH) appear at 8.54, 7.53, 7.46, 7.40, 7.11, 7.07, 6.85 and 6.74 ppm, –CH protons in appear at 6.52, 6.21, 5.90 and 5.50 and –CH_3_ protons appear at 3.17, 3.11, 2.68, 2.22, 1.72 and 1.27 ppm, which supports the conclusions reached by the FT-IR results. Our research laboratory is currently working on the design of chromogenic biosensors based on cyanine dyes with better solubility in aqueous media by replacing the chlorine group with a high capacity electron-donating or withdrawing groups (such as methyl, nitro, methoxyl and benzo in increasing order) and/or binding with nucleic acids in the heptamethine bridge [16,17]. The results of these investigation will be reported in due course.

## 4. Conclusions

IR-786, a heptamethine cyanine dye was demonstrated to be a highly selective and sensitive sensor for cyanide anions in water–MeCN mixtures and solvent-free solid surfaces. IR-786 exhibited three unique properties: broad spectral range, and a large hypsochromic (blue) shift of about 345 nm (from 775 nm to 430 nm) in the presence of cyanide anions. The mechanism for the binding of cyanide and hydroxide anions to IR-786 was elucidated using FT-IR and NMR spectroscopy, where cyanide and hydroxide are thought to bind to different carbons on the IR-786 backbone. The most probable mechanism is the nucleophilic addition of CN^−^ to the indolium group of IR-786, as supported by the observed changes in absorption spectrum. The use of IR-786 in a solvent-free solid state sensor for the selective sensing of CN^−^ in the environment was demonstrated, where CN^−^ in the range of 50–300 μM in water samples with minimal interference by OH^−^ was detected using colorimetric and fluorescence based detection methods.

## Supplementary Materials

The following are available online at http://www.mdpi.com/1424-8220/16/3/271/s1, Figure S1: Color changes of IR-786 (50 μM) under normal light and under hand held UV lamp (λ = 365 nm) in MeCN: water mixtures = 75%:25%, 50%:50%, 25%:75% before and after the addition of various anions. Figure S2: (**a**) UV-vis absorption spectra and (**b**) absorbance ratiometric values (absorbance value at 430 divided by absorbance value at 775 nm) before and after the addition of 1.0 equivalence of various anions to IR-786 in 75%:25% MeCN–water mixture (50 μM) and (**c**) emission spectra and (**d**) emission ratiometric values (intensity value at 520 nm divided by intensity value at 800 nm) obtained from (**a**). Figure S3: (**a**) UV-vis absorption spectra of IR-786 in 75%:25% MeCN–water mixture (50 μM) before and after the addition of CN^−^ (up to 8.0 μM) and (**b**) absorbance ratiometric values (absorbance value at 430 divided by absorbance value at 775 nm) obtained from (**c**) Fluorescence emission spectra (λex = 430 nm, excitation slit = 20 nm, emission slit = 20 nm), for IR-786 (5.0 μM) 75%:25% MeCN–water mixture before and after the addition of OH^−^ (up to 10 μM) and (**d**) fluorescence emission ratiometric values (intensity value at 520 nm divided by intensity value at 800 nm) obtained from (**c**). Arrows show the direction of increased amount of anions. Figure S4: Job’s plots for the determination of the binding stoichiometry between IR-786 (5.0 μM) and CN^−^ in (**a**) 100% MeCN (**b**) 75%:25% MeCN–H_2_O mixture based on absorbance at λmax = 430 nm and (**c**) 100% MeCN (**d**) 75%:25% MeCN–H_2_O mixture based on fluorescence emission at λmax = 520 nm. Figure S5: Job’s plot for the determination of the binding stoichiometry between IR-786 (5.0 μM) and OH^−^ in 100% MeCN based on absorbance at λmax = 430 nm. Figure S6: Plots for the determination of the binding constants between IR-786 (50 μM) and CN^−^ in (**a**) 100% MeCN (**b**) 75%:25% MeCN–H_2_O mixture based on absorbance at λmax = 430 nm and (**c**) 100% MeCN (**d**) 75%:25% MeCN–H_2_O mixture based on fluorescence emission at λmax = 520 nm. Figure S7: Change in pH of IR-786 solution in MeCN during the titration of stock solution of CN− and OH− in buffer as described in the experimental section. Volume of anions correspond to 0–20 μM.

## Abbreviations

The following abbreviations are used in this manuscript:

IR 786: IR 786 perchlorate or 2-(2-[2-Chloro-3-([1,3-dihydro-1,3,3-trimethyl-2*H*-indol-2-ylidene]ethylidene)-1-cyclohexen-1-yl]ethenyl)-1,3,3-trimethylindolium perchlorate
MeCN: Acetonitrile
