# Supplementary material for: A Highly Selective Sensor for Cyanide in Organic Media and on Solid Surfaces
Source: Sensors (Basel). 2016 Feb 24;16(3):271. doi: 10.3390/s16030271 (PMC4813846; doi:10.3390/s16030271)
Supplement: Supplementary file 1 [file sensors-16-00271-s001.docx]

Supplementary Materials: A Highly Selective Sensor for Cyanide in Organic Media and on
Solid Surfaces

Belygona Barare, Ilknur Babahan, Yousef M. Hijji, Enock Bonyi, Solomon Tadesse and Kadir Aslan


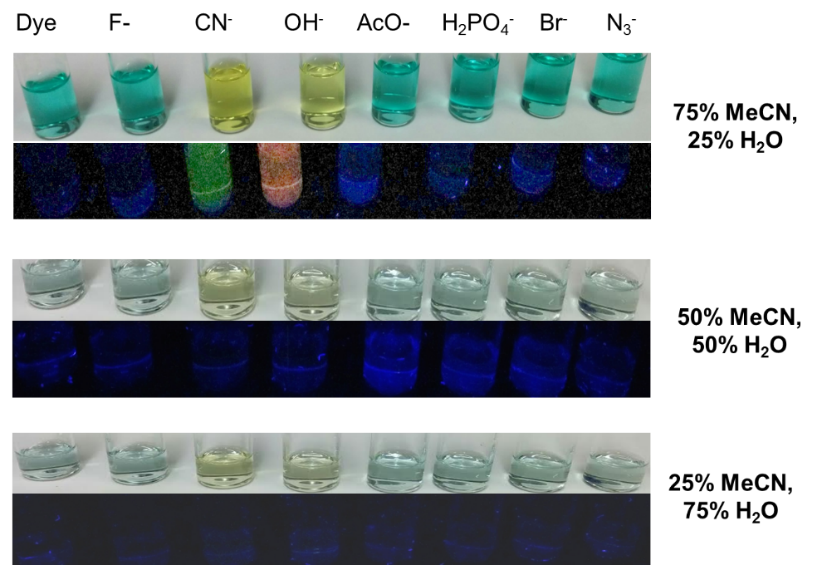


**Figure S1.** Color changes of IR-786 (50 μM) under normal light and under hand held UV lamp (λ = 365 nm) in MeCN: water mixtures = 75%:25%, 50%:50%, 25%:75% before and after the addition of various anions.


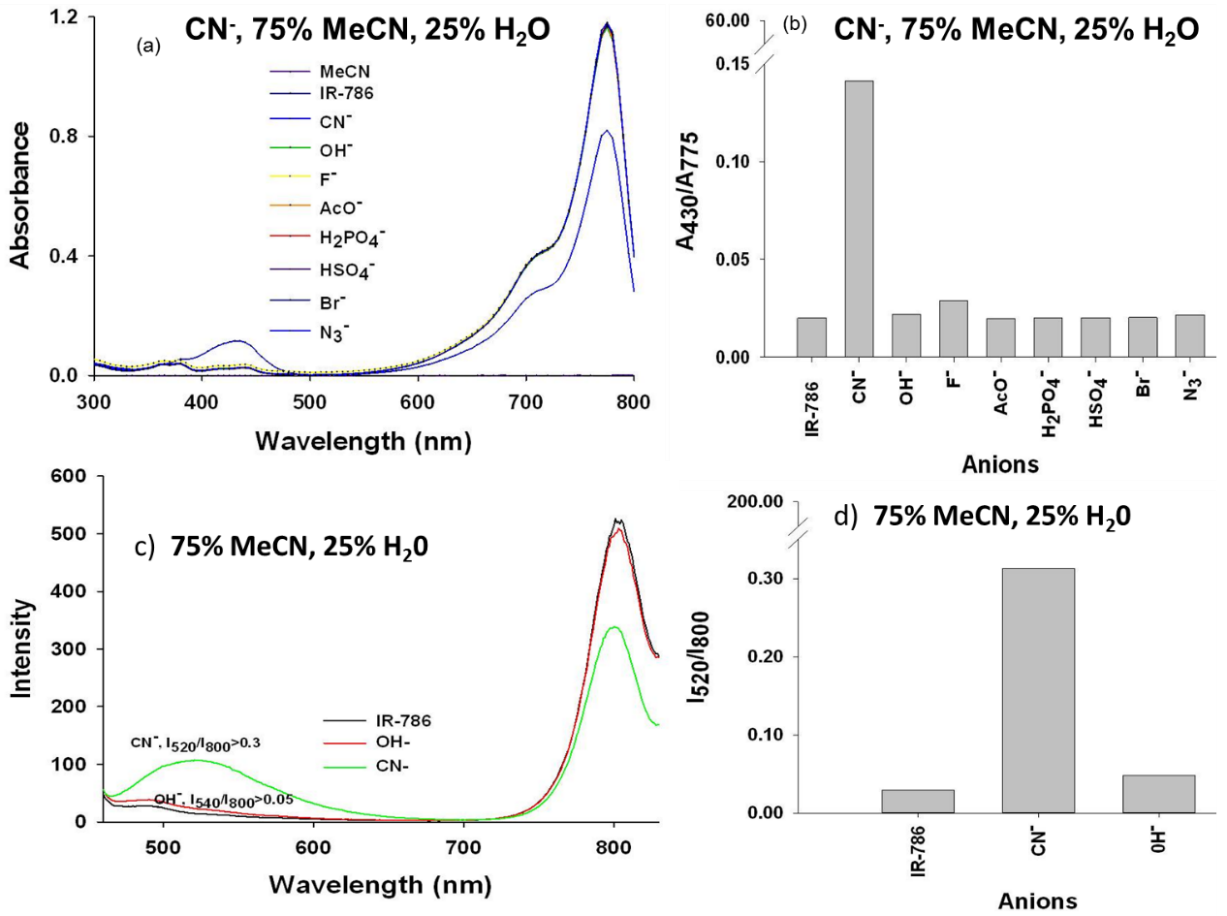


**Figure S2.** (**a**) UV-vis absorption spectra and (**b**) absorbance ratiometric values (absorbance value at 430 divided by absorbance value at 775 nm) before and after the addition of 1.0 equivalence of various anions to IR-786 in 75%:25% MeCN:water mixture (50 μM) and (**c**) emission spectra and (**d**) emission ratiometric values (intensity value at 520 nm divided by intensity value at 800 nm) obtained from (**a**).


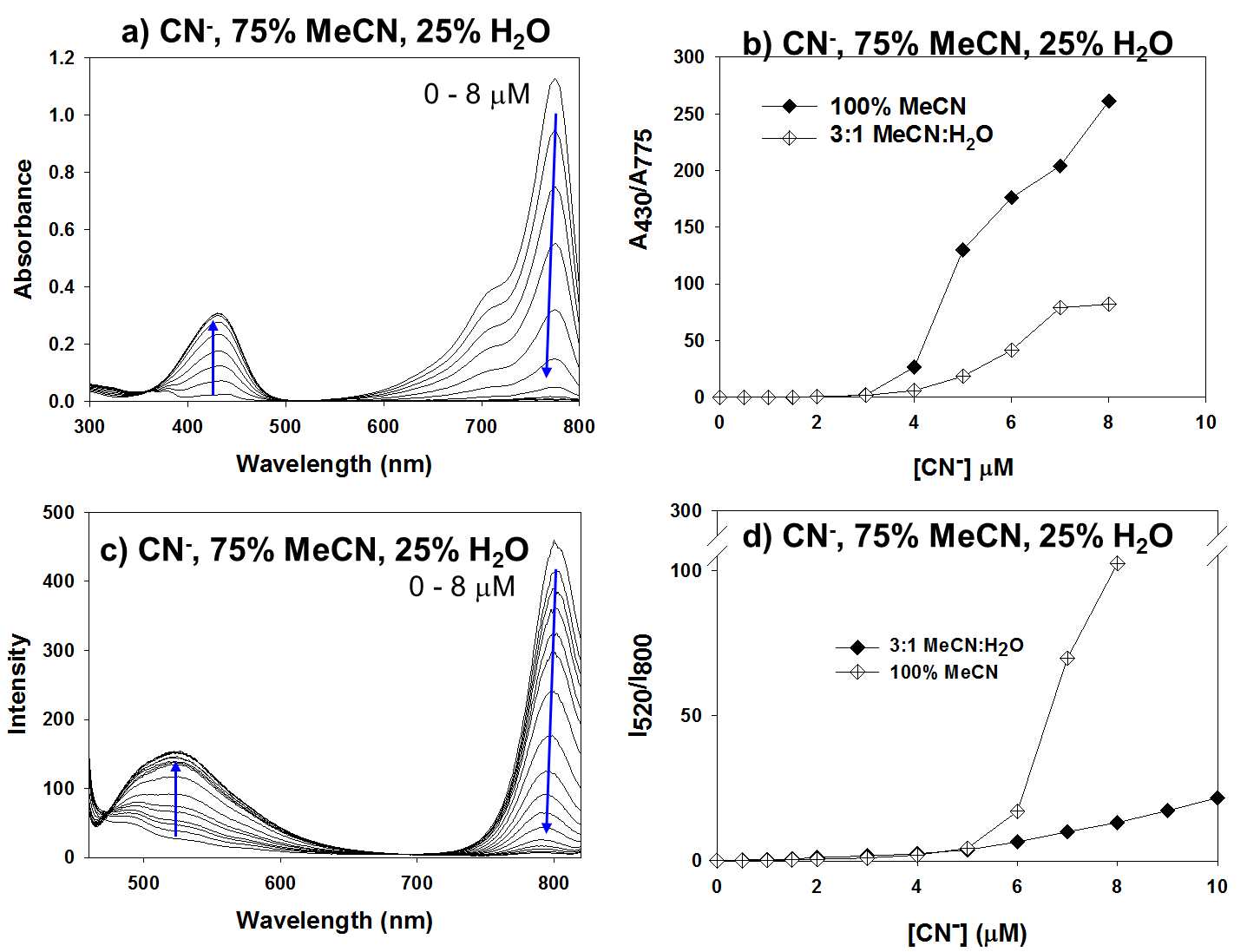


**Figure S3.** (**a**) UV-vis absorption spectra of IR-786 in 75%:25% MeCN:water mixture (50 μM) before and after the addition of CN^−^ (up to 8.0 μM) and (**b**) absorbance ratiometric values (absorbance value at 430 divided by absorbance value at 775 nm) obtained from (**c**) Fluorescence emission spectra
(λ_ex_ = 430 nm, excitation slit = 20 nm, emission slit = 20 nm), for IR-786 (5.0 μM) 75%:25% MeCN:water mixture before and after the addition of OH^−^ (up to 10 μM) and (**d**) fluorescence emission ratiometric values (intensity value at 520 nm divided by intensity value at 800 nm) obtained from (**c**). Arrows show the direction of increased amount of anions.


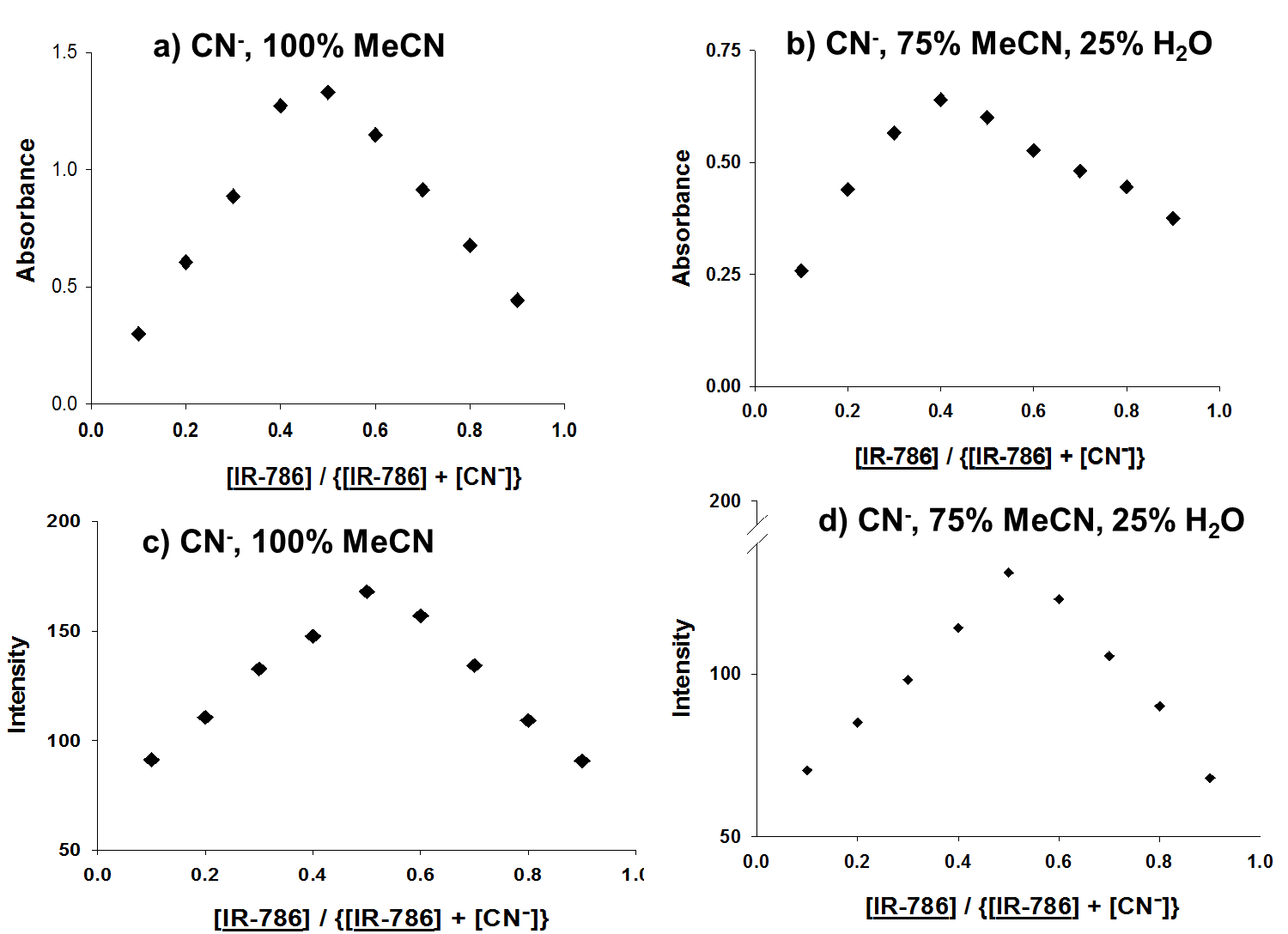


**Figure S4.** Job’s plots for the determination of the binding stoichiometry between IR-786 (5.0 μM) and CN^−^ in (**a**) 100% MeCN (**b**) 75%:25% MeCN:H_2_O mixture based on absorbance at λ_max_ = 430 nm and (**c**) 100% MeCN (**d**) 75%:25% MeCN:H_2_O mixture based on fluorescence emission at λ_max_ = 520 nm.


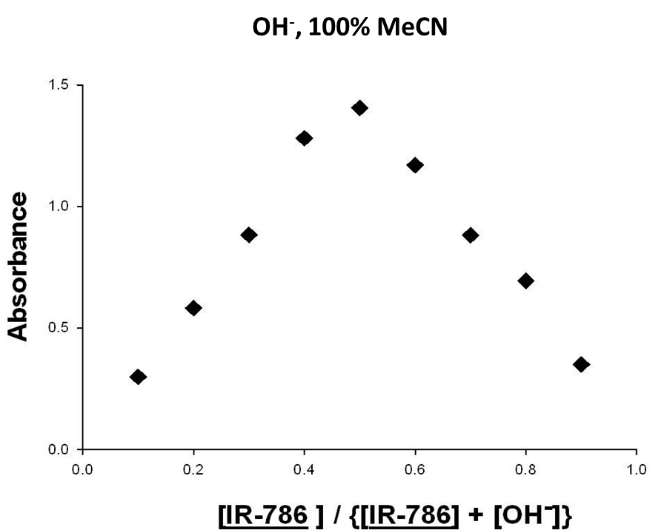


**Figure S5.** Job’s plot for the determination of the binding stoichiometry between IR-786 (5.0 μM) and OH^−^ in 100% MeCN based on absorbance at λ_max_ = 430 nm.


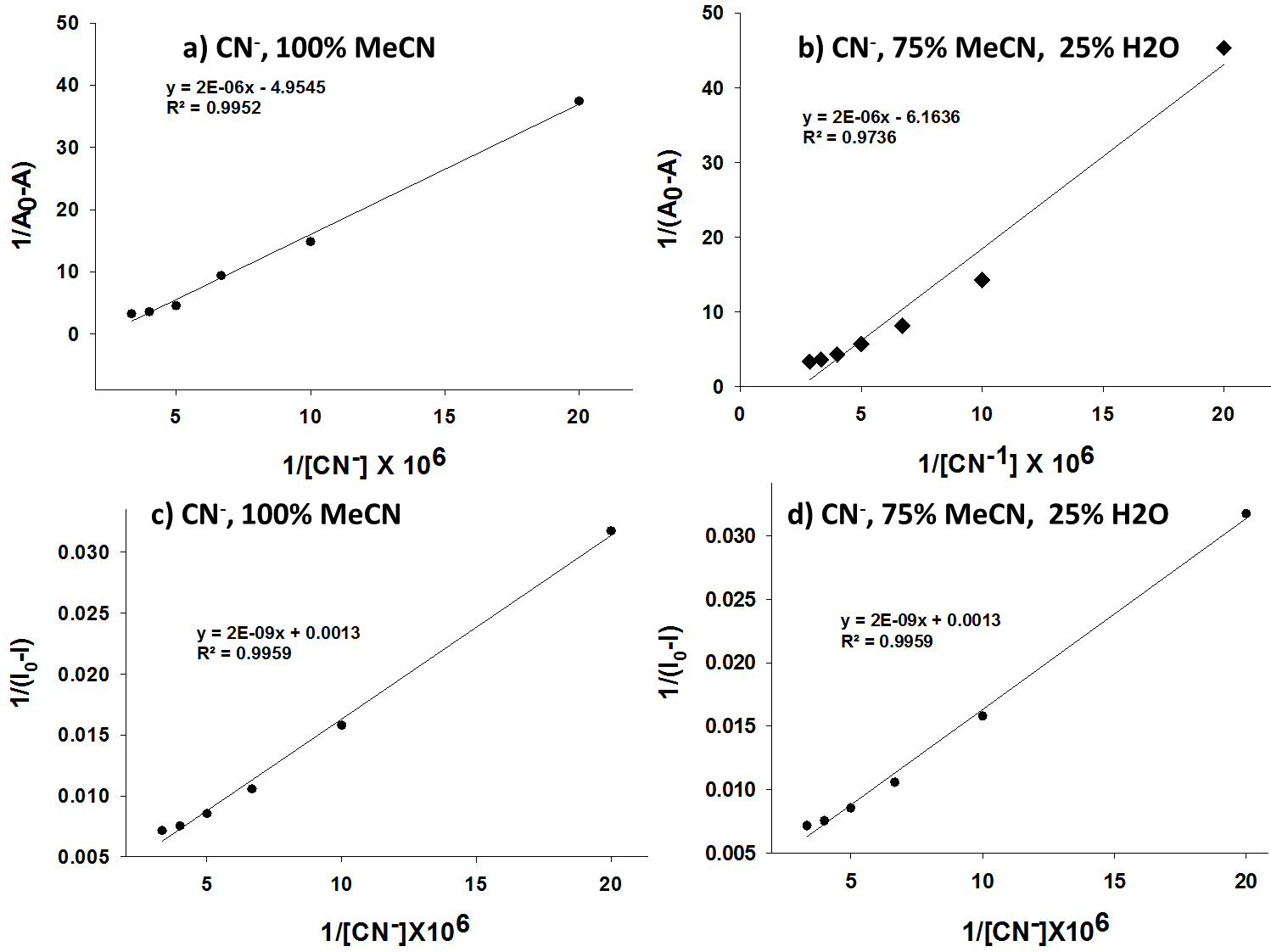


**Figure S6.** Plots for the determination of the binding constants between IR-786 (50 μM) and CN^−^ in (**a**) 100% MeCN (**b**) 75%:25% MeCN:H_2_O mixture based on absorbance at λ_max_ = 430 nm and (**c**) 100% MeCN (**d**) 75%:25% MeCN:H_2_O mixture based on fluorescence emission at λ_max_ = 520 nm.

**Figure S7.** Change in pH of IR-786 solution in MeCN during the titration of stock solution of CN^−^ and OH^−^ in buffer as described in the experimental section. Volume of anions correspond to 0–20 μM.
